# (*E*)-2-[(2-Hydroxy­ethyl)iminiometh­yl]-6-methoxy­phenolate

**DOI:** 10.1107/S1600536809005297

**Published:** 2009-02-21

**Authors:** Guo-Xia Tan, Xi-Cheng Liu

**Affiliations:** a Experimental & Educational Technology Center, Linyi Normal University, Linyi 276005, People’s Republic of China; b Department of Chemistry, Qufu Normal University, Qufu 273165, People’s Republic of China

## Abstract

The title Schiff base compound, C_10_H_13_NO_3_, obtained by the reaction of 2-hydr­oxy-3-methoxy­benzaldehyde and 2-amino­ethanol in methanol solution, crystallizes in a zwitterionic form, in which the mol­ecule adopts a *trans* configuration about the central C=N bond. An intra­molecular N—H⋯O hydrogen bond occurs. In the crystal structure, mol­ecules are linked into chains by inter­molecular O—H⋯O hydrogen bonding.

## Related literature

For related structures, see: Cui *et al.* (1999 ▶); Dong *et al.* (2007 ▶); Li *et al.* (2005 ▶); Ng (2008 ▶); Oshio *et al.* (2003 ▶); Sun *et al.* (2006 ▶). For reference structural data, see: Allen *et al.* (1987 ▶).
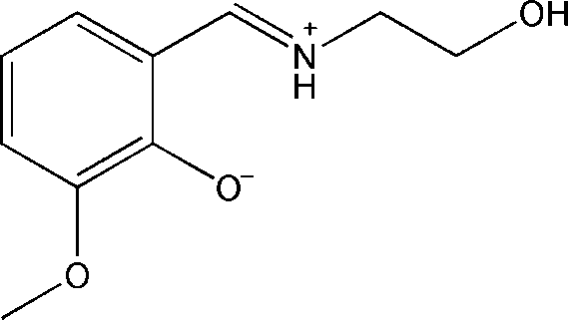


## Experimental

### 

#### Crystal data

C_10_H_13_NO_3_
*M* *_r_* = 195.21
Orthorhombic, 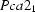

*a* = 14.148 (6) Å
*b* = 6.587 (3) Å
*c* = 10.760 (4) Å
*V* = 1002.8 (7) Å^3^
*Z* = 4
Mo *K*α radiation
μ = 0.10 mm^−1^
*T* = 295 K
0.30 × 0.30 × 0.12 mm

#### Data collection

Bruker SMART APEX CCD diffractometer
Absorption correction: multi-scan (*SADABS*; Bruker, 2002 ▶) *T*_min_ = 0.974, *T*_max_ = 0.991
7345 measured reflections
1041 independent reflections
923 reflections with *I* > 2σ(*I*)
*R*_int_ = 0.037

#### Refinement

*R*[*F* ^2^ > 2σ(*F* ^2^)] = 0.034
*wR*(*F* ^2^) = 0.084
*S* = 1.07
1041 reflections
127 parameters
1 restraint
H-atom parameters constrained
Δρ_max_ = 0.09 e Å^−3^
Δρ_min_ = −0.15 e Å^−3^

### 

Data collection: *SMART* (Bruker, 2002 ▶); cell refinement: *SAINT* (Bruker, 2002 ▶); data reduction: *SAINT*; program(s) used to solve structure: *SHELXS97* (Sheldrick, 2008 ▶); program(s) used to refine structure: *SHELXL97* (Sheldrick, 2008 ▶); molecular graphics: *ORTEP-3 for Windows* (Farrugia, 1997 ▶); software used to prepare material for publication: *SHELXL97*.

## Supplementary Material

Crystal structure: contains datablocks global, I. DOI: 10.1107/S1600536809005297/hb2911sup1.cif

Structure factors: contains datablocks I. DOI: 10.1107/S1600536809005297/hb2911Isup2.hkl

Additional supplementary materials:  crystallographic information; 3D view; checkCIF report

## Figures and Tables

**Table 1:** Hydrogen-bond geometry (Å, °)

*D*—H⋯*A*	*D*—H	H⋯*A*	*D*⋯*A*	*D*—H⋯*A*
N1—H1⋯O1	0.86	1.95	2.617 (2)	134
O3—H3⋯O1^i^	0.82	1.95	2.741 (3)	161

Symmetry code: (i) 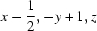.

**Fig. 1.**
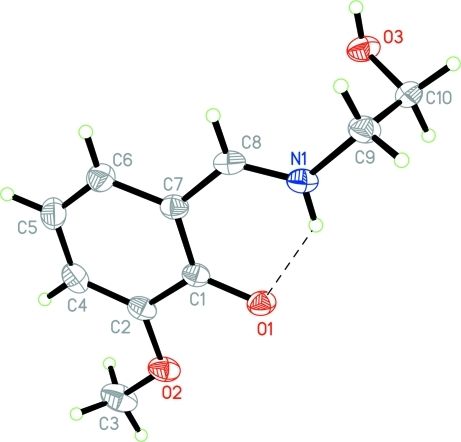
The structure of (I) with displacement ellipsoids drawn at the 50% probability level. The N—H···O hydrogen bond is shown as a dashed line.

## supplementary crystallographic information

### Comment

The title compound, (I), derived from 3-methoxy-2-hydroxybenzaldehyde and 2-aminoethanol, is a potential NO_3_ tetradentate Schiff base ligand and its complexes with Cd(II), Cu(II), Zn(II) and Fe(III) have been reported (Cui *et al.*, 1999; Dong *et al.*, 2007; Li *et al.*, 2005; Oshio *et al.*, 2003). Here, the structure of (I) is described.

The title molecule exists in a zwitterionic form with a strong intramolecular N—H···O hydrogen bond (Table 1) between the NH^+^ and the phenolate O^-^, as shown in Fig. 1. The bond lengths and angles are within normal ranges (Allen *et al.*,1987). The N1=C8 [1.294 (3) Å] and N1—C8 [1.453 (3) Å] bond distances are comparable to these found in similar Schiff base compounds, such as 2,4-Dibromo-6-(2-hydroxyethyliminiomethyl)- phenolate [1.277 (5) and 1.451 (4) Å] (Sun *et al.*, 2006) and 4-chloro-2-[tris(hydroxymethyl)methyliminiomethyl]phenolate [1.288 (2) and 1.467 (2) Å] (Ng, 2008). As expected, the molecule adopts a *trans* configuration about the central C=N bond. In the crystal structure, O3—H3···O1^i^ (symmetry code as given in Table 1) intermolecular hydrogen bonds formed between the hydroxy and oxygen of phenolate link the molecules into a one-dimension supramolecular chain.

### Experimental

3-Methoxy-2-hydroxybenzaldehyde (0.152 g, 1 mmol) and equimolar 2-aminoethanol (0.061 g, 1 mmol) were refluxed for 30 min in methanol solution (15 ml). The reaction mixtures were cooled to room temprature and filtered. After keeping the filtrate in air for 3 d, yellow blocks of (I) (yield 66%; mp 338–339 K) were obtained.

### Refinement

H atoms were placed at calculated positions and refined in the riding-model approximation, with C—H = 0.93 Å and *U*_iso_(H) = 1.2Ueq(C) for *sp*^2^ H atoms, C—H = 0.96 Å and *U*_iso_(H) = 1.5Ueq(C) for methyl H atoms, C—H = 0.97 Å and *U*_iso_(H) = 1.2Ueq(C) for methylene H atoms, N—H = 0.86 Å and *U*_iso_(H) = 1.2Ueq(C) for imino group, and O—H = 0.82 Å and *U*_iso_(H) = 1.5Ueq(C) for hydroxy. Friedel pairs were merged.

### Crystal data

**Table:**

C_10_H_13_NO_3_	*F*(000) = 416
*M**_r_* = 195.21	*D*_x_ = 1.293 Mg m^−^^3^
Orthorhombic, *P**c**a*2_1_	Mo *K*α radiation, λ = 0.71073 Å
Hall symbol: P 2c -2ac	Cell parameters from 1828 reflections
*a* = 14.148 (6) Å	θ = 2.9–20.4°
*b* = 6.587 (3) Å	µ = 0.10 mm^−^^1^
*c* = 10.760 (4) Å	*T* = 295 K
*V* = 1002.8 (7) Å^3^	Block, yellow
*Z* = 4	0.30 × 0.30 × 0.12 mm

### Data collection

**Table:**

Bruker APEX CCD diffractometer	1041 independent reflections
Radiation source: fine-focus sealed tube	923 reflections with *I* > 2σ(*I*)
graphite	*R*_int_ = 0.037
φ and ω scans	θ_max_ = 26.0°, θ_min_ = 2.9°
Absorption correction: multi-scan (*SADABS*; Bruker, 2002)	*h* = −17→17
*T*_min_ = 0.974, *T*_max_ = 0.991	*k* = −8→8
7345 measured reflections	*l* = −12→13

### Refinement

**Table:**

Refinement on *F*^2^	Primary atom site location: structure-invariant direct methods
Least-squares matrix: full	Secondary atom site location: difference Fourier map
*R*[*F*^2^ > 2σ(*F*^2^)] = 0.034	Hydrogen site location: inferred from neighbouring sites
*wR*(*F*^2^) = 0.084	H-atom parameters constrained
*S* = 1.07	*w* = 1/[σ^2^(*F*_o_^2^) + (0.0473*P*)^2^ + 0.0337*P*] where *P* = (*F*_o_^2^ + 2*F*_c_^2^)/3
1041 reflections	(Δ/σ)_max_ < 0.001
127 parameters	Δρ_max_ = 0.09 e Å^−^^3^
1 restraint	Δρ_min_ = −0.15 e Å^−^^3^

### Special details

**Table:**

Geometry. All e.s.d.'s (except the e.s.d. in the dihedral angle between two l.s. planes) are estimated using the full covariance matrix. The cell e.s.d.'s are taken into account individually in the estimation of e.s.d.'s in distances, angles and torsion angles; correlations between e.s.d.'s in cell parameters are only used when they are defined by crystal symmetry. An approximate (isotropic) treatment of cell e.s.d.'s is used for estimating e.s.d.'s involving l.s. planes.
Refinement. Refinement of *F*^2^ against ALL reflections. The weighted *R*-factor *wR* and goodness of fit *S* are based on *F*^2^, conventional *R*-factors *R* are based on *F*, with *F* set to zero for negative *F*^2^. The threshold expression of *F*^2^ > σ(*F*^2^) is used only for calculating *R*-factors(gt) *etc*. and is not relevant to the choice of reflections for refinement. *R*-factors based on *F*^2^ are statistically about twice as large as those based on *F*, and *R*- factors based on ALL data will be even larger.

### Fractional atomic coordinates and isotropic or equivalent isotropic displacement parameters (Å^2^)

**Table:**

	*x*	*y*	*z*	*U*_iso_*/*U*_eq_	
C1	0.53278 (16)	0.7444 (3)	0.0860 (2)	0.0408 (5)	
C2	0.57651 (16)	0.9338 (3)	0.0535 (2)	0.0451 (6)	
C3	0.69788 (18)	1.1717 (4)	0.1022 (3)	0.0718 (9)	
H3A	0.6519	1.2763	0.1164	0.108*	
H3B	0.7207	1.1807	0.0183	0.108*	
H3C	0.7497	1.1885	0.1588	0.108*	
C4	0.5396 (2)	1.0535 (4)	−0.0390 (3)	0.0557 (7)	
H4	0.5680	1.1776	−0.0566	0.067*	
C5	0.4599 (2)	0.9927 (5)	−0.1078 (3)	0.0625 (8)	
H5	0.4366	1.0750	−0.1710	0.075*	
C6	0.41744 (18)	0.8135 (4)	−0.0814 (3)	0.0570 (7)	
H6	0.3655	0.7718	−0.1278	0.068*	
C7	0.45145 (15)	0.6891 (4)	0.0160 (2)	0.0436 (5)	
C8	0.39971 (16)	0.5130 (4)	0.0485 (2)	0.0460 (6)	
H8	0.3479	0.4790	−0.0006	0.055*	
C9	0.36073 (17)	0.2262 (4)	0.1804 (3)	0.0535 (6)	
H9A	0.4005	0.1091	0.1961	0.064*	
H9B	0.3173	0.1915	0.1140	0.064*	
C10	0.30532 (16)	0.2758 (4)	0.2962 (3)	0.0529 (6)	
H10A	0.2717	0.1553	0.3232	0.064*	
H10B	0.3490	0.3137	0.3617	0.064*	
N1	0.41934 (14)	0.3958 (3)	0.1415 (2)	0.0478 (5)	
H1	0.4701	0.4199	0.1829	0.057*	
O1	0.56592 (11)	0.6322 (2)	0.17429 (18)	0.0486 (4)	
O2	0.65545 (11)	0.9784 (3)	0.1213 (2)	0.0564 (5)	
O3	0.23996 (12)	0.4345 (2)	0.2785 (2)	0.0584 (5)	
H3	0.1946	0.3924	0.2387	0.088*	

### Atomic displacement parameters (Å^2^)

**Table:**

	*U*^11^	*U*^22^	*U*^33^	*U*^12^	*U*^13^	*U*^23^
C1	0.0358 (11)	0.0387 (12)	0.0478 (13)	0.0046 (9)	0.0063 (10)	−0.0074 (11)
C2	0.0393 (12)	0.0407 (13)	0.0553 (16)	0.0030 (10)	0.0127 (11)	−0.0106 (12)
C3	0.0623 (17)	0.0421 (15)	0.111 (3)	−0.0143 (14)	0.0175 (19)	−0.0154 (16)
C4	0.0608 (17)	0.0435 (14)	0.0629 (17)	0.0038 (13)	0.0206 (15)	0.0050 (13)
C5	0.0670 (18)	0.0703 (19)	0.0502 (16)	0.0090 (15)	0.0064 (14)	0.0132 (15)
C6	0.0507 (14)	0.0726 (18)	0.0477 (15)	0.0022 (13)	−0.0017 (12)	0.0014 (14)
C7	0.0378 (12)	0.0484 (13)	0.0445 (13)	0.0014 (11)	0.0028 (10)	−0.0071 (12)
C8	0.0341 (12)	0.0521 (14)	0.0520 (14)	0.0004 (10)	−0.0026 (11)	−0.0117 (12)
C9	0.0420 (13)	0.0424 (13)	0.0760 (18)	−0.0020 (10)	−0.0002 (14)	0.0002 (13)
C10	0.0422 (12)	0.0533 (15)	0.0633 (16)	0.0033 (11)	−0.0071 (12)	0.0138 (13)
N1	0.0354 (10)	0.0481 (12)	0.0600 (14)	−0.0036 (9)	−0.0025 (9)	−0.0050 (11)
O1	0.0400 (8)	0.0440 (9)	0.0619 (11)	−0.0040 (7)	−0.0082 (8)	0.0003 (9)
O2	0.0436 (9)	0.0433 (9)	0.0822 (14)	−0.0083 (8)	0.0020 (9)	−0.0062 (9)
O3	0.0410 (9)	0.0591 (10)	0.0750 (13)	0.0088 (9)	−0.0069 (9)	−0.0038 (10)

### Geometric parameters (Å, °)

**Table:**

C1—O1	1.291 (3)	C6—H6	0.9300
C1—C7	1.423 (3)	C7—C8	1.416 (3)
C1—C2	1.436 (3)	C8—N1	1.294 (3)
C2—O2	1.366 (3)	C8—H8	0.9300
C2—C4	1.373 (4)	C9—N1	1.453 (3)
C3—O2	1.422 (3)	C9—C10	1.508 (4)
C3—H3A	0.9600	C9—H9A	0.9700
C3—H3B	0.9600	C9—H9B	0.9700
C3—H3C	0.9600	C10—O3	1.409 (3)
C4—C5	1.407 (4)	C10—H10A	0.9700
C4—H4	0.9300	C10—H10B	0.9700
C5—C6	1.355 (4)	N1—H1	0.8600
C5—H5	0.9300	O3—H3	0.8200
C6—C7	1.415 (4)		
			
O1—C1—C7	122.5 (2)	C6—C7—C1	121.3 (2)
O1—C1—C2	121.3 (2)	C8—C7—C1	119.8 (2)
C7—C1—C2	116.2 (2)	N1—C8—C7	124.6 (2)
O2—C2—C4	125.1 (2)	N1—C8—H8	117.7
O2—C2—C1	114.1 (2)	C7—C8—H8	117.7
C4—C2—C1	120.8 (2)	N1—C9—C10	111.6 (2)
O2—C3—H3A	109.5	N1—C9—H9A	109.3
O2—C3—H3B	109.5	C10—C9—H9A	109.3
H3A—C3—H3B	109.5	N1—C9—H9B	109.3
O2—C3—H3C	109.5	C10—C9—H9B	109.3
H3A—C3—H3C	109.5	H9A—C9—H9B	108.0
H3B—C3—H3C	109.5	O3—C10—C9	113.0 (2)
C2—C4—C5	121.5 (2)	O3—C10—H10A	109.0
C2—C4—H4	119.2	C9—C10—H10A	109.0
C5—C4—H4	119.2	O3—C10—H10B	109.0
C6—C5—C4	119.5 (3)	C9—C10—H10B	109.0
C6—C5—H5	120.2	H10A—C10—H10B	107.8
C4—C5—H5	120.2	C8—N1—C9	124.0 (2)
C5—C6—C7	120.6 (3)	C8—N1—H1	118.0
C5—C6—H6	119.7	C9—N1—H1	118.0
C7—C6—H6	119.7	C2—O2—C3	117.4 (2)
C6—C7—C8	118.8 (2)	C10—O3—H3	109.5
			
O1—C1—C2—O2	1.6 (3)	C2—C1—C7—C6	1.1 (3)
C7—C1—C2—O2	−178.65 (19)	O1—C1—C7—C8	4.8 (3)
O1—C1—C2—C4	−178.8 (2)	C2—C1—C7—C8	−175.0 (2)
C7—C1—C2—C4	1.0 (3)	C6—C7—C8—N1	−174.5 (2)
O2—C2—C4—C5	177.5 (2)	C1—C7—C8—N1	1.8 (3)
C1—C2—C4—C5	−2.1 (3)	N1—C9—C10—O3	63.7 (3)
C2—C4—C5—C6	1.0 (4)	C7—C8—N1—C9	174.2 (2)
C4—C5—C6—C7	1.2 (4)	C10—C9—N1—C8	−104.5 (3)
C5—C6—C7—C8	174.0 (2)	C4—C2—O2—C3	5.8 (3)
C5—C6—C7—C1	−2.2 (4)	C1—C2—O2—C3	−174.6 (2)
O1—C1—C7—C6	−179.1 (2)		

### Hydrogen-bond geometry (Å, °)

**Table:**

*D*—H···*A*	*D*—H	H···*A*	*D*···*A*	*D*—H···*A*
N1—H1···O1	0.86	1.95	2.617 (2)	134
O3—H3···O1^i^	0.82	1.95	2.741 (3)	161

Symmetry codes: (i) *x*−1/2, −*y*+1, *z*.
